# R7 Photoreceptor Specification in the Developing Drosophila Eye: The Role of the Transcription Factor Deadpan

**DOI:** 10.1371/journal.pgen.1006159

**Published:** 2016-07-18

**Authors:** Yannis Emmanuel Mavromatakis, Andrew Tomlinson

**Affiliations:** Department of Genetics and Development, College of Physicians and Surgeons, Columbia University, New York, New York, United States of America; New York University, UNITED STATES

## Abstract

As cells proceed along their developmental pathways they make a series of sequential cell fate decisions. Each of those decisions needs to be made in a robust manner so there is no ambiguity in the state of the cell as it proceeds to the next stage. Here we examine the decision made by the Drosophila R7 precursor cell to become a photoreceptor and ask how the robustness of that decision is achieved. The transcription factor Tramtrack (Ttk) inhibits photoreceptor assignment, and previous studies found that the RTK-induced degradation of Ttk was critically required for R7 specification. Here we find that the transcription factor Deadpan (Dpn) is also required; it is needed to silence *ttk* transcription, and only when Ttk protein degradation and transcriptional silencing occur together is the photoreceptor fate robustly achieved. Dpn expression needs to be tightly restricted to R7 precursors, and we describe the role played by Ttk in repressing *dpn* transcription. Thus, Dpn and Ttk act as mutually repressive transcription factors, with Dpn acting to ensure that Ttk is effectively removed from R7, and Ttk acting to prevent Dpn expression in other cells. Furthermore, we find that N activity is required to promote *dpn* transcription, and only in R7 precursors does the removal of Ttk coincide with high N activity, and only in this cell does Dpn expression result.

## Author Summary

Animals are made from a vast diversity of different cell types, and understanding how they are specified is a major goal of developmental biology. In this study we use the Drosophila R7 photoreceptor as a model system for understanding how cell fate specification occurs. We examine the step when the R7 precursor cell adopts the photoreceptor fate, and ask how the signaling pathways active in the cell are integrated to provide an unambiguous directive to become a photoreceptor. The transcription factor Tramtrack (Ttk) represses the ability of the cell to become a photoreceptor, and how it is removed is the focus of this study. Previous work identified a protein degradation mechanism, and here we describe the role of the transcription factor Deadpan (Dpn) in repressing *ttk* transcription. We find that both the protein degradation mechanism and transcriptional silencing are required for efficient Ttk removal. Dpn expression needs to be restricted to the R7 precursor and we describe how the mutual antagonism between Ttk and Dpn and the action of the Notch signaling pathway are integrated to ensure that Dpn is selectively expressed in the cell.

## Introduction

Development proceeds in a stepwise manner in which cells pass through a series of interim states in the progression from a pluripotent precursor to a fully-specified and final cell fate. Positional signals play a prominent role in this mechanism, with the receipt of the signals promoting the cells to the next state in the series. Each interim state needs to be established robustly so that upon receipt of the subsequent round of signals, the advance to the next state is unambiguous. In this paper we use the R7 photoreceptor of the developing *Drosophila* eye as a model cell with which to study the molecular mechanisms that occur in the acquisition of interim cell states. Specifically we examine the choice made by the R7 precursor to become a photoreceptor and the mechanisms that cooperate and consolidate that choice.

**Fig 1:**
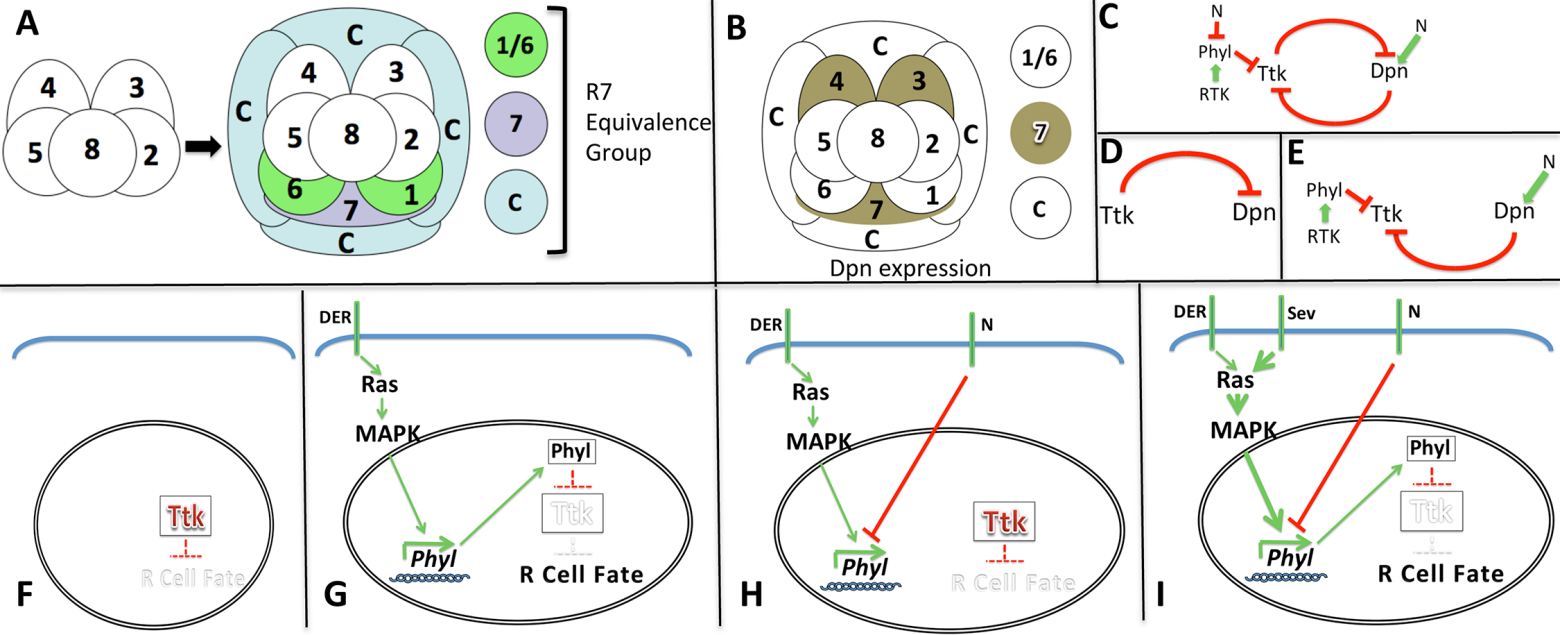
Schematic summaries of the R7 equivalence group, the Dpn expression pattern, and the signaling pathways that regulate Ttk degradation. (**A**) The precluster is made from prospective photoreceptors R2,3,4,5,8 (open white circles). It is the foundation cluster to which cells are recruited and then specified to their fate. The first seven cells added are the R7 equivalence group; R7, R1/6 and four cone cells (c). R7 and R1/6 are photoreceptors, but cone cells are non-photoreceptors. (**B**) Dpn is expressed in the R3/4 cells of the precluster, and is then expressed in R7 and not the other cells of the equivalence group [12]. (**C**) The epistatic relationship of the factors controlling the Ttk/Dpn interaction. The role of N in repressing Phyl is inferred (see H below). (**D**) In the early R7 precursor *ttk* transcription is active and there are high levels of Ttk which repress *dpn* transcription, and Dpn is absent from the cell. (**E**) As the RTK pathway degrades Ttk, the block to *dpn* transcription is released and the high N activity promotes gene expression leading to Dpn protein which prevents further transcription from the *ttk* locus. (**F**) A naïve R7 equivalence group cell expresses Ttk, and whether that Ttk is degraded or not determines whether the photoreceptor fate is specified. (**G**) In R1/6 precursors DER activates the RTK pathway that leads to Phyl expression and the consequential Ttk degradation. (**H**) In R7 equivalence cells with high N activity (R7 and the cone cells) there is a block to the DER induced degradation of Ttk. The block here is indicated as preventing *phyl* transcription. This is our current hypothesis, and is used here as a simple visual devise to indicate the block. (**I**) In R7 (and not cone cell) precursors, the RTK pathway is boosted by the activation of Sev. This boost is sufficient to overcome the block imposed by N and to achieve the degradation of Ttk. Since this Sev boost to the RTK pathway does not occur in cone cells they remain in the state shown in H with Ttk not degraded.

The R7 precursor belongs to a group of cells known as the R7 equivalence group, which gives rise (in addition to R7) to the two R1/6 photoreceptors and the four (non-photoreceptor) cone cells. The fates of the equivalence group cells are determined by the positions they occupy in the growing ommatidial cluster (Fig 1A), and each initially expresses the transcription factor Tramtrack (Ttk88—hereafter referred to as Ttk) which represses the photoreceptor fate. If a cell eliminates Ttk then it becomes a photoreceptor; if not it becomes a cone cell [1, 2].

Removal of Ttk is achieved by the activation of the RTK pathway which induces the transcription of *phyllopod* (*phyl*) [3, 4], a gene encoding an adaptor protein that promotes the Ubiquitination and degradation of Ttk [5] (Fig 1G).

The degradation of Ttk in R1/6 precursors is achieved in a relatively simple manner; activation of the RTK pathway by the Drosophila EGF Receptor (DER) removes Ttk (Fig 1G). But the process in the R7 precursor is more complicated because this cell also experiences high N activity which antagonizes the RTK function (Fig 1H), and a much stronger RTK signal is needed in this cell for Ttk removal. This is supplied by the Sevenless (Sev) RTK [7] (Fig 1I) which is expressed at high levels in R7 precursors, and is activated by its ligand Bride of Sevenless (Boss) presented on the adjacent R8 cell [8]. Previously, it was assumed that the Sev-boosted RTK signal simply overcame the N antagonism and promoted the levels of Phyl required for Ttk degradation. In this paper we find that, in addition to the expression of Phyl, a concurrent suppression of *ttk* transcription is also required for R7 photoreceptor specification, and this is achieved by the action of the Deadpan (Dpn) protein.

Dpn is a transcription factor of the helix-loop-helix class [9], closely related to the E(Spl) proteins, and behaves as a direct N response gene in neuroblasts [10] and in the eye [11]. Dpn is expressed in R7 precursors, but not in any other cells of the R7 equivalence group [12] (Fig 1B). This feature led us to investigate the roles it plays in the specification of this cell. In this work, we not only determine that Dpn is required to repress *ttk* transcription specifically in R7 precursors, but we also establish that ectopic Dpn disturbs the fates of the other cells of the R7 equivalence group, and we infer that Dpn expression needs to be tightly restricted to the R7 precursor. This restriction is established by a two-tier regulation of its gene transcription. First, Ttk represses *dpn* transcription (in the nascent R7 precursor Ttk levels are high and *dpn* transcription is repressed). However, as the action of the RTK pathway degrades Ttk, that repression is lost. Second, high N activity is required to promote *dpn* transcription, and the only cell of the R7 equivalence group that degrades Ttk and has high N activity is R7.

Dpn and Ttk act here as mutually repressive transcription factors (Fig 1C), and this relationship lies at the heart of the mechanisms used to silence *ttk* transcription and to restrict Dpn expression to the R7 precursor. In the nascent R7, Ttk is dominant in the relationship with active transcription and high protein levels, while *dpn* transcription is repressed and no protein is present (Fig 1D). But once the RTK pathway becomes active this relationship inverts. First Ttk protein is degraded, removing the repression on *dpn* transcription, and the high N activity then drives *dpn* gene expression with the resulting Dpn protein acting to inhibit *ttk* transcription. Thus at the end of the mechanism, Dpn is dominant; with active transcription and high protein levels, whilst *ttk* transcription is silenced and no protein is present (Fig 1E).

Dpn action is required in R7 precursors because the high N activity opposes Ttk degradation. Even with the use of Sev to provide a potent RTK signal in the cell, the degradation mechanism is not able to robustly specify R7 precursors; Dpn repression of *ttk* transcription is also required. The degradation mechanism removes extant protein, and the transcriptional regulation prevents protein resupply, and the two combine to specify the photoreceptor fate in the R7 precursor.

## Results

### Phyl and Dpn synergize in R7 specification

#### Phyl expression is insufficient for robust R7 specification

**Fig 2:**
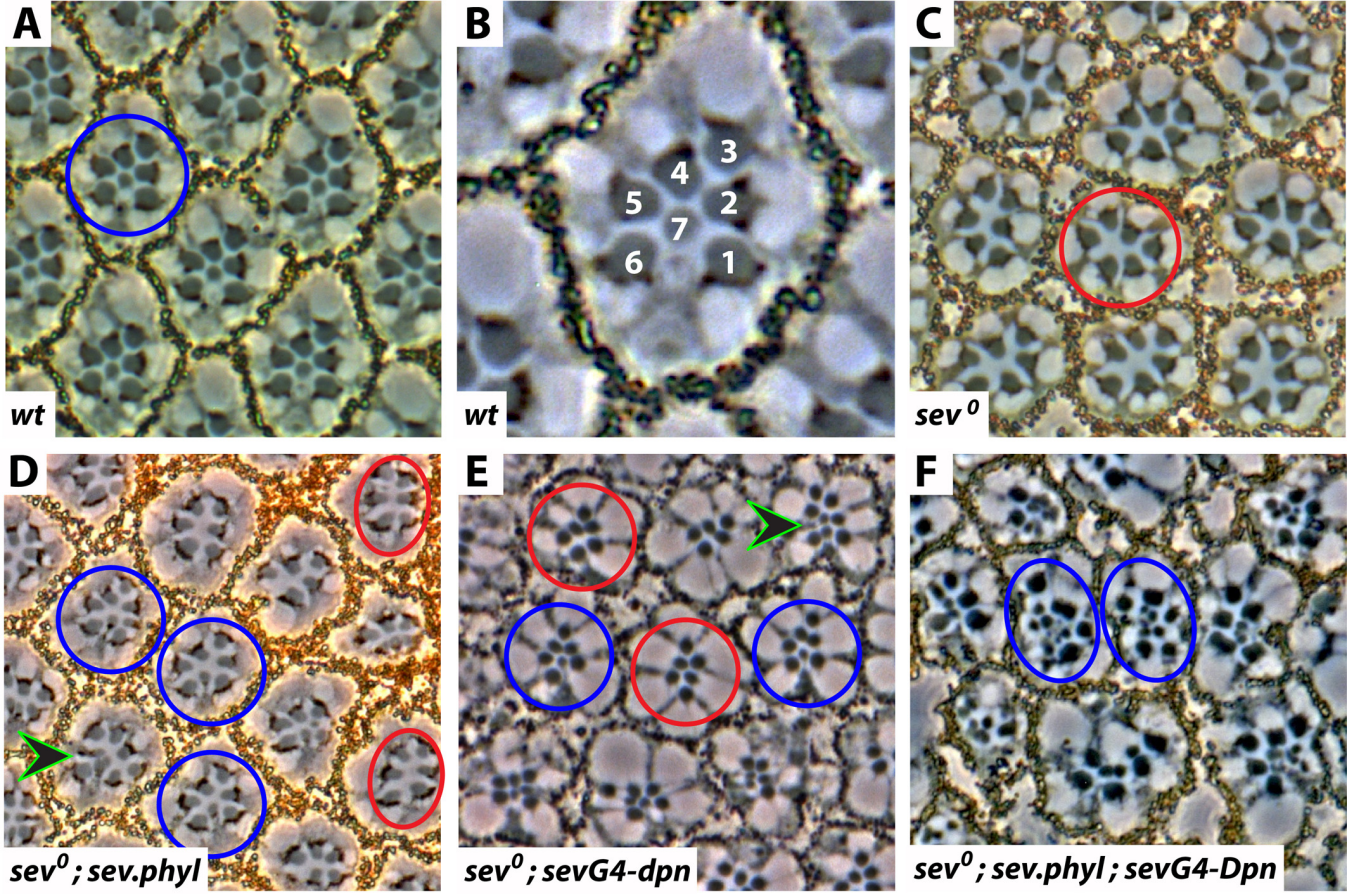
Dpn and Phyl synergize in R7 specification. (**A**) Section through the apical regions of a wild type eye showing the ommatidial array. The blue circle circumscribes the photoreceptors of a single ommatidium. (**B**) High power image of an ommatidium shown in A. The rhabdomeres (grey circles) of the six outer photoreceptor (R1-6) form an asymmetric trapezoidal shape surrounding the small rhabdomere of the R7 photoreceptor. (**C**) Section through the apical regions of a *sev°* eye in which each ommatidium (red circle) contains only the R1-6 outer photoreceptors. (**D**) When *sev*.*phyl* is expressed in a *sev°* eye many ommatidia have rescued R7s (blue circles) but many do not (red circles). In addition to the rescue of native R7s, ectopic R7s are also observed (arrowhead). (**E**) When *sevG4-dpn* is expressed in a *sev°* eye, R7s are rescued in some ommatidia (blue circles) and some ectopic R7s are formed (arrowhead), but the majority of ommatidia lack R7s (red circles). (**F**) When *sev*.*phyl* and *sevG4-dpn* are expressed together in a *sev°* eye there is a potent induction of R7s. Ommatidia now contain many R7s (blue circles), indicating a powerful synergy between Phyl and Dpn expression in rescuing the absence of Sev.

Models of R7 photoreceptor specification have for some time seen the RTK-induced expression of Phyl as the critical step in R7 specification. We too considered Phyl expression essential, but also realized that experiments had not been performed to determine whether Phyl expression alone was sufficient for R7 photoreceptor specification. We therefore inactivated high-level RTK signaling in R7 precursors by removing Sev (*sev°)*, and then used a *sev*.*phyl* transgene (in which *phyl* is expressed under *sev* transcriptional control) to resupply *phyl* transcription to that background (*sev°; sev*.*phyl*). Sections through adult eyes of this genotype showed three types of ommatidia (Fig 2D); those in which R7s were not rescued (and ommatidia appeared as in *sev°*—red circles), those in which R7s were rescued (blue circles), and those in which ectopic R7 were present (arrowhead) that were likely derived from cone cell precursors. The majority of ommatidia showed rescued R7s with and without the ectopic types, but many still lacked R7s. In this experiment, multimerized *sev* enhancer sequences are used [13], and in our hands this provides a potent level of transcription to the R7 precursor. The absence of a Phyl antibody prevented us from examining the levels of Phyl expression, and we therefore doubled the transgene number (*sev°; 2Xsev*.*phyl*) but still observed many ommatidia lacking R7s (21%, N = 324), and we therefore infer that simple expression of Phyl in R7 precursors does not fully rescue the absence of Sev. This argues that something other than Phyl expression is required for robust R7 specification.

#### Dpn synergizes with Phyl in R7 specification

We had begun working on Dpn because of its R7-restricted expression, and wondered whether it may be required along with Phyl for robust R7 specification. To test this we drove expression of a *UAS*.*dpn* transgene with *sev*.*Gal4* (*sevG4-dpn*) in a *sev* mutant background, and observed that 35% (N = 347) of ommatidia had rescued R7s whether native (blue circles) or ectopic types (arrowhead) while 65% lacked R7s (red circles—Fig 2E).

This argues that Dpn and Phyl share similar properties in rescuing *sev°* R7s, albeit with Dpn appearing the weaker of the two in this ability. When we combined the expressions of Dpn and Phyl in the absence of Sev (*sev°; UAS*.*dpn; sev*.*Gal4/sev*.*phyl*) sections through adult eyes showed multiple R7s in all ommatidia, indicating the rescue of native R7s and the transformation of multiple cone cell precursors (blue circles—Fig 2F). Thus, there is a dramatic synergy between Phyl and Dpn in their ability to rescue R7 in the absence of Sev, suggesting that Dpn may represent the factor required along with Phyl for the robust specification of the R7 fate. This led us to perform a detailed analysis of the roles and expression patterns of Dpn.

### Dpn is required for robust R7 specification

To determine the roles Dpn plays in R7 specification, we undertook an investigation of its loss-of-function phenotypes.

#### Analysis of *dpn* mutant eye clones

**Fig 3:**
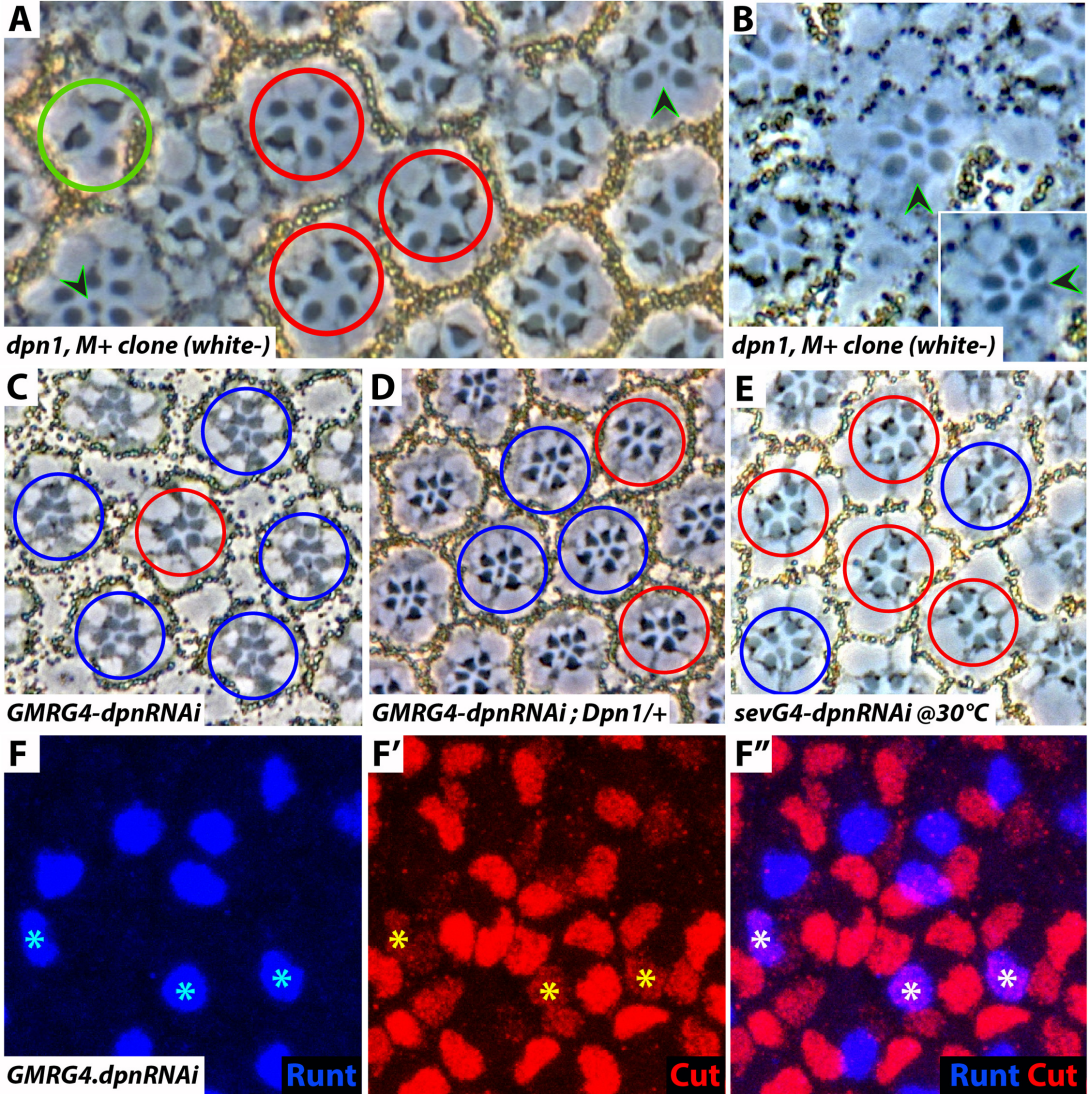
Dpn loss-of-function phenotypes. (**A**) Section through a *dpn1*, *Minute*^*+*^ clone. The mutant cells lack pigment. Red circles indicate ommatidia lacking R7s, and arrowheads point to mutant cells forming normal R7s. The green circle highlights an ommatidium with only two large rhabdomere cells. (**B**) Section through a *dpn1*, *Minute*^*+*^ clone showing an ommatidium in which all photoreceptors lack the *dpn* gene but still possess an R7 (arrowhead). The inset shows the same ommatidium at a deeper level showing the R8 also lacking pigment (arrowhead). (**C**) Section though a *GMRG4-dpnRNAi* eye. A low frequency of ommatidia lack R7s (red circle). (**D**) When a copy of the *dpn* gene is removed in the *GMRG4-dpnRNAi* background there is an increase in the number of ommatidia lacking R7s (red circles). (**E**) Section through a *sevG4-dpnRNAi* eye raised at 30°C, many ommatidia lack R7s (red circles). (**F, F’,F”**) Confocal image of *GMRG4-dpnRNAi* developing ommatidial clusters in which cells in the R7 position (asterisks) can be seen expressing both an R7 marker (Runt–blue) and a cone cell marker (Cut–red).

To examine the effects of removing *dpn* function, we induced clones (marked by the loss of the pigment gene *white*) in adult eyes. These clones were very small and present only in the anterior parts of the eyes, suggesting that homozygous *dpn1* cells are out-competed by the surrounding wild type tissue. We repeated this experiment but also gave the mutant clones a *Minute*^*+*^ competitive advantage and allowed the survival of the mutant tissue. In these *dpn1* mutant patches many ommatidia lacked R7s (Fig 3A, red circles), but in other ommatidia R7’s were specified normally even though those R7s were mutant for *dpn* (lacking pigment—arrowheads). Dpn is expressed in R3/4 and R7 precursors and we asked whether perhaps, it was the R3/4 expression that was needed for R7 specification rather than an autonomous function in the R7 precursor. To address this we performed mosaic analysis, and found that no cell, or group of cells, was obligatorily required to carry *dpn*^*+*^ gene function for the formation of normal (R7-carrying) ommatidia. Indeed, Fig 3B shows a normal R7-carrying ommatidium (arrowhead), in which all photoreceptors are *dpn* mutant (lacking pigment) including R8 (arrowhead in inset). In addition to these R7-related phenotypes we also observed ommatidia containing dramatically reduced photoreceptor number (Fig 3A, green circle) and ommatidia in which a supernumerary large rhabdomere cell was present. These features likely result from the functions of Dpn in the early ommatidium, and since they do not relate to the mechanisms of R7 specification, we do not investigate them further in this paper.

#### The effects of dpnRNAi transgenes

We next examined the effects of knocking down *dpn* gene function using *RNAi*. When a *UAS*.*dpnRNAi* transgene was driven by *GMR*.*Gal4* (which expresses in a blanket manner in the eye disc), infrequent ommatidia lacking R7s (1.3%, N = 608) were observed in adult eyes (Fig 3C–red circle). When a copy of the *wild type* gene was also removed in this background the frequency of ommatidia lacking R7s increased to 16%, (N = 101) (Fig 3D–red circles). This enhancement suggests that these RNAi phenotypes result from specific knockdown of Dpn function (as opposed to off-target effects). The *Gal4/UAS* system is temperature sensitive, and when the *UAS*.*dpnRNAi* line was driven by *sev*.*Gal4* at 30°C, 22% of ommatidia (N = 480) lacked R7s (Fig 3E—red circles).

Both clonal analysis and RNAi experiments reveal a clear but not fully penetrant loss of R7s when *dpn* function is removed/abrogated. From this we infer that R7 precursors lacking *dpn* gene function lie on the cusp of adopting or not adopting the photoreceptor fate.

#### *dpn* mutant R7s show ambiguous cell fate markers

The loss of R7 characteristically occurs when the RTK pathway is insufficiently activated in the R7 precursor. Under this circumstance, Ttk is not degraded and the cell is specified as a non-photoreceptor cone cell. To determine whether the failure to generate R7s in *dpn* mutant ommatidia results from their transformation to cone cells, we examined *GMRG4-dpnRNAi* third instar developing ommatidia. In mutations such as *sev*, in which RTK activity is severely compromised, there is a "clean" switch from the R7 to the cone cell fate [14], and all cells in the R7 position express the cone cell marker Cut, and do not express the R7 marker Runt. However, when we examined *GMRG4-dpnRNAi* developing ommatidia we did not see such clean transformations. Rather, ommatidia had cells in the R7 positions expressing both Cut and Runt, indicating a hybrid R7/cone cell identity (Fig 3F). Since in this genotype few adult ommatidia showed a loss of R7s, we infer that the majority of these “hybrid" R7s become the normal R7s evident in the adult eyes, and the minority adopt the cone cell fate leading to the ommatidia lacking R7s.

#### Ectopic expression of Dpn induces supernumerary R7s

We next returned to the effects of ectopic Dpn expression that we had cursorily examined when we investigated its synergistic interaction with Phyl.

**Fig 4:**
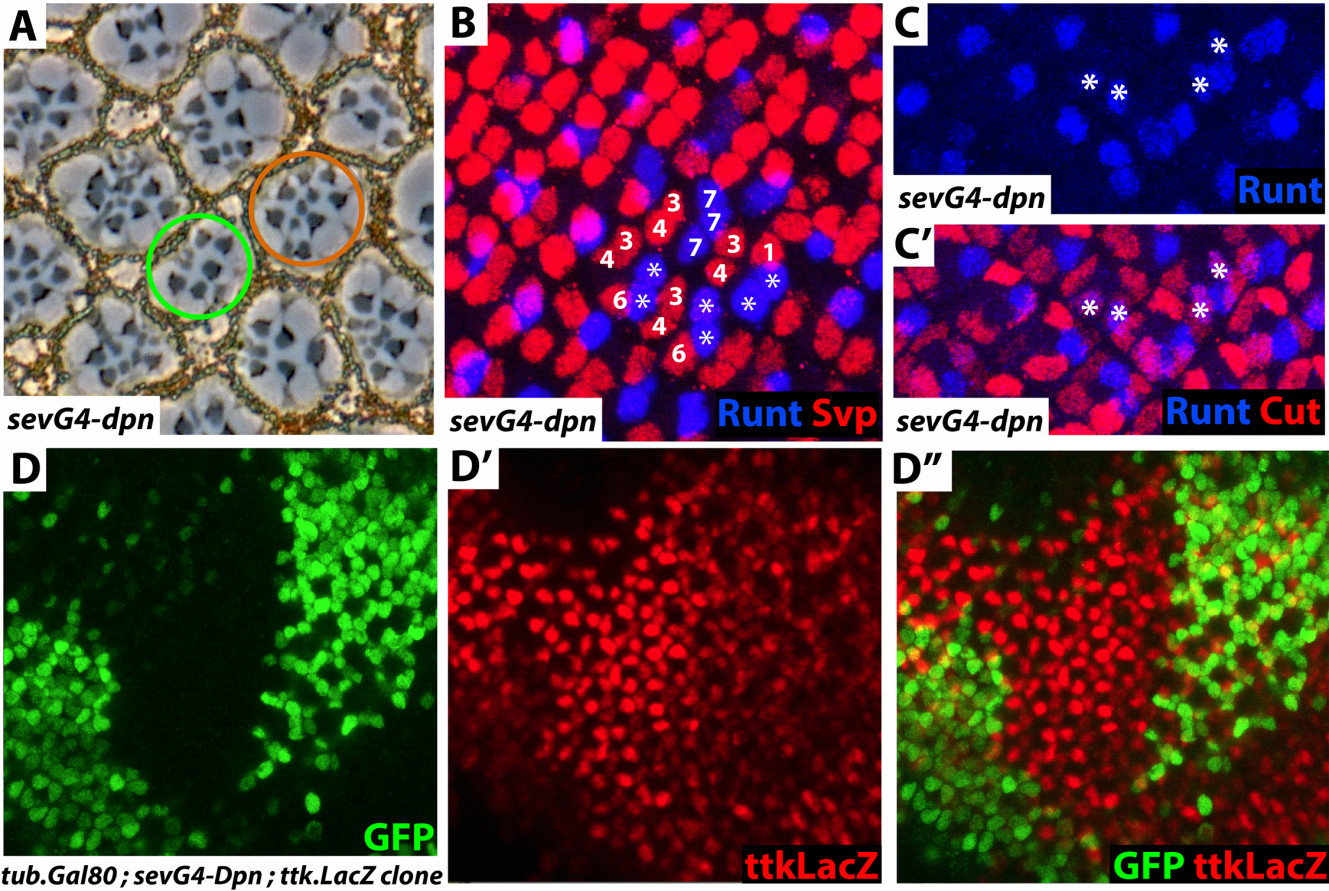
Dpn expressions converts R1/6 and cone cell precursors into R7s, and suppresses *ttk* transcription. (**A**) Section through a *sevG4-dpn* eye. Some ommatidia show ectopic R7s characteristically derived from cone cells (brown circle), others have ectopic R7s that appear to be derived from R1/6 precursors (green circle) while other ommatidia may be a mixture of the two. (**B**) Confocal image of *sevG4-dpn* eye disc stained for Svp (red–marker of the R3/4 and R1/6 cell types), and Runt (blue–R7 marker). In some ommatidia three cells occupy the R1/6/7 positions and all three (numbered) express Runt, but in other ommatidia only two cells are present in this position and both express Runt (asterisks). (**C, C’**) Images of *sev*.*G4-dpn* eye discs examining more mature ommatidia, in which cells in the cone cell positions frequently express both the cone cell marker Cut (red) and the R7 marker Runt (blue) (asterisks). (**D,D’,D”**) Confocal image of clones in which a *tub*.*Gal80 flip-out cassette* is excised allowing for *sevG4-dpn* expression (green). Where Dpn is expressed there is a dramatic reduction in cone cell expression of *ttk*.*lacZ* (red).

*sevG4-dpn adult analysis*. *sev*.*Gal4* is expressed at high levels in all R7 equivalence group cells (R1/6, R7 and cone cells) and we used it to drive expression of *UAS*.*dpn* (hereafter *sevG4-dpn*). Sections through such *sevG4-dpn* adult eyes showed ommatidia containing extra R7-like cells. 81% of 235 ommatidia showed one or more supernumerary R7s (Fig 4A). Supernumerary R7s can arise from R1/6 or cone cell precursors. When they are derived from R1/6 precursors, two or three R7s cluster together in the R1/6/7 position, but when derived from cone cells they project into the center of the ommatidia from eccentric positions. The supernumerary R7s in *sevG4-dpn* eyes appeared to be derived from both sources; with the green circle indicating an ommatidium with R1/6-derived R7s, and the brown circle highlighting an ommatidium with apparent cone-cell derived supernumerary R7s. Other ommatidia appeared as a combination of these two types, and we surmised that both R1/6 and cone cell precursors gave rise to R7 cells in *sevG4-dpn* eyes.

*sevG4-dpn eye disc analysis*. We next examined *sevG4-dpn* eye discs, and indeed observed evidence for the transformations of both R1/6 (Fig 4B) and cone cells precursors (Fig 4C) into R7 types. The transformations of these two different precursors pools likely result from two distinctly different effects of Dpn. When Ttk is removed from a presumptive photoreceptor, the presence of high N activity specifies that cell as an R7 rather than the R1/6 type [6], and we infer that Dpn mimics this N function, transforming R1/6 precursors into R7s. The focus of this paper is the role played by Dpn in achieving Ttk elimination, and since the mechanism that transforms the R1/6 precursors is unrelated to this, we do not pursue this result any further. In the Discussion we revisit this point and discuss it in detail. Examination of cone cell precursors in *sevG4-dpn* eye discs (Fig 4C) revealed them to show a similar hybrid identity similar to that displayed by *dpn* mutant R7s; they expressed both the R7 marker (Runt) and the cone cell marker (Cut). When strong activators of the RTK pathway are expressed in cone cell precursors their transformation into R7 types is vigorous, and hybrid cells expressing both R7 and cone cell markers are rarely observed. Hence, the hybrid identity of the cells in the *sevG4-dpn* eye discs suggests that a potent transformation does not occur. However, since there are many cone-cell-derived R7s apparent in the adult eyes, we infer that a significant fraction of these cells eventually differentiate as R7s.

#### Dpn represses *ttk* transcription

The results to this point indicated that Dpn function was intimately related to photoreceptor specification, and we wondered whether it acted to regulate *ttk* transcription. We could not perform preliminary experiments to assess whether Dpn expression regulates levels of Ttk protein since we no longer have a useful antibody. Instead, we moved immediately to examine Dpn effects on the *ttk*.*lacZ* reporter line. *ttk*.*lacZ* is expressed at high levels in the cone cell precursors (and at lower levels in the basal pool of non-cluster cells) and we examined whether ectopic Dpn expression (*sevG4-dpn*) in cone cell precursors would change their level of *ttk*.*lacZ* expression. In this experiment we excised a *tub*.*Gal80* transgene in clonal patches in the *sev*.*Gal4;UAS*.*dpn/ttk*.*lacZ* background. In the presence of Gal80, the *Sev*.*Gal4* transgene is unable to drive *UAS*.*dpn* expression. Once *Gal80* is excised, Dpn expression ensues. We stained third instar eye discs with anti-Dpn to indicate the cells in which the excision had occurred, and observed a clear and correlating down-regulation of *ttk*.*lacZ* expression in the cone cells without affecting the expression in the basal pool (Fig 4D). Hence, ectopic expression of Dpn in cone cells strongly reduces the activity of the *ttk* transcriptional reporter, and we infer that a native function of Dpn is to reduce/eliminate *ttk* transcription from R7 precursors.

### The role of Ttk in repressing Dpn expression

**Fig 5:**
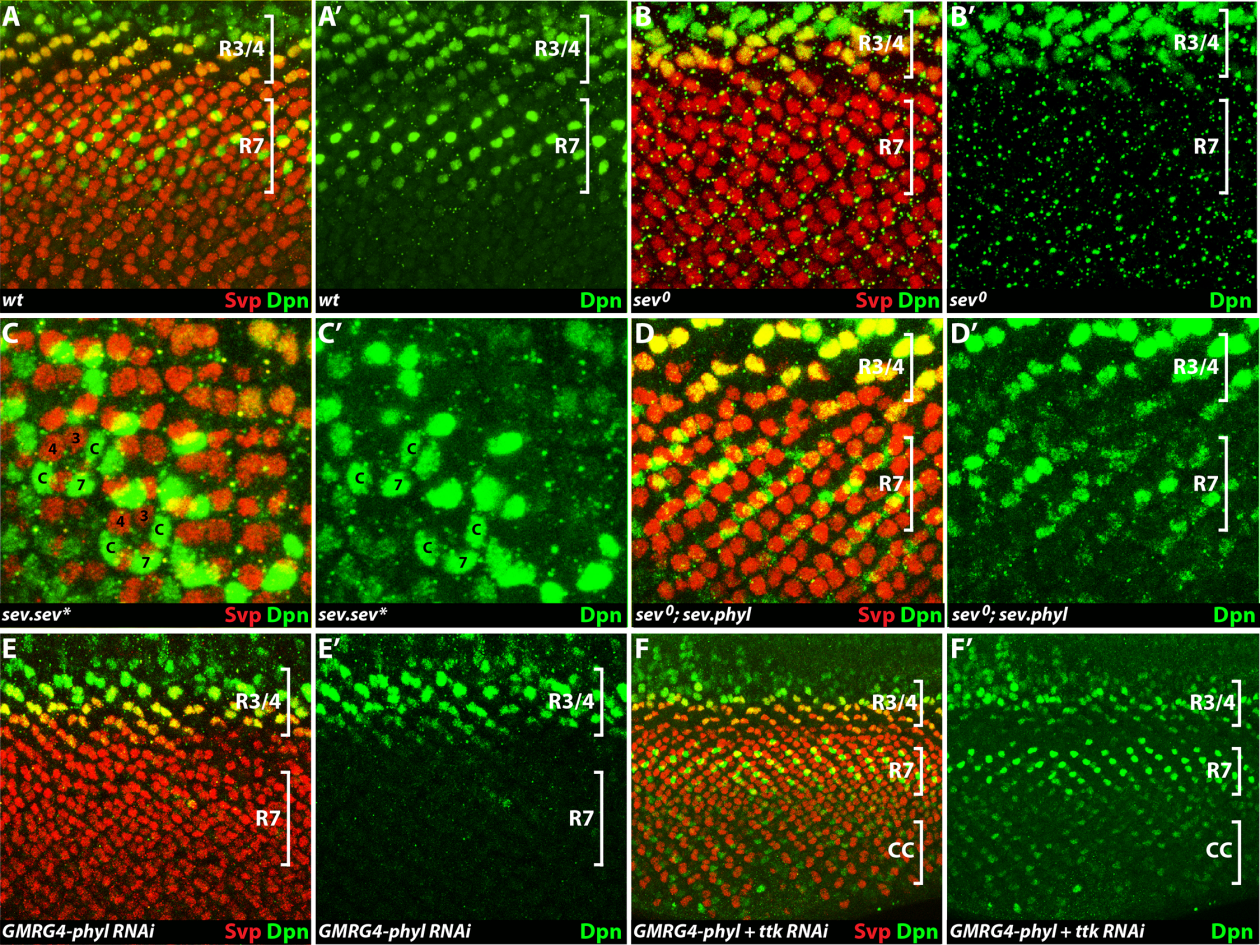
RTK pathway regulation of Dpn expression. In each panel Dpn is stained in green, and Svp (red) is used as a counterstain to highlight the R3/4 and R1/6 precursors. (**A, A’**) In a wild type eye disc Dpn expression occurs in three phases. First, it is expressed in unidentified cells in the morphogenetic furrow (above the indicated R3/4 staining). Second, it is expressed in the R3/4 precursors (upper bracket) as evidenced by their co-expression of Svp. Third, it is expressed in R7 precursors (that do not express Svp) (lower bracket). (**B, B’**) In *sev°* eye discs Dpn expression is selectively missing from R7 precursors. (**C, C’**) In *sev*.sev* eye discs, activated Sev is expressed in cone cell precursors (*c*) and they express Dpn. (**D’, D’**). R7 Dpn expression is restored in *sev°* by the presence of *sev*.*phyl*. (**E, E’**) UAS.*phylRNAi* driven by *GMR*.*Gal4* removes Dpn expression from R7 but not R3/4 precursors. (**F, F’**) Dpn expression is restored in R7 precursors when *ttkRNAi* is expressed concomitantly with *phylRNAi*. Additionally, Dpn staining is now also seen in cone cell (cc) precursors (lower bracket).

When Dpn is ectopically expressed in R1/6 or cone cell precursors, these cells are re-specified as R7 types (Fig 4A–4C), which highlights the need to restrict Dpn expression in the R7 equivalence group to the R7 precursor. We therefore investigated the mechanisms that restricts Dpn expression, and began with a fine-detailed analysis of its expression pattern. Dpn is first expressed in unidentified cells of very early ommatidial clusters, and then becomes expressed in the R3/4 precluster cells (top bracket Fig 5A'). As the second-wave-cells are added to the cluster, the expression decays in R3/4, and Dpn is then expressed in R7 precursors (lower bracket Fig 5A'). This R7 expression is striking in that it is early and ephemeral, and is lost from the cell before any neuronal or R7-specific markers are expressed. This suggested that Dpn expression is likely controlled by the signals that act on the early R7 precursor, and the RTK and N pathways were the most likely candidates. In this section we examine how the RTK pathway and the removal of Ttk control Dpn expression, and in later sections we address the role played by N.

#### Sev is required for Dpn expression

We asked whether high-level RTK activity is required for R7 Dpn expression by removing *sev* gene function. In *sev°* eye discs, Dpn protein is expressed normally in the early clusters and in the R3/4 cells but is absent from R7 precursors (Fig 5B), suggesting that Sev activity is critically required for the expression of Dpn in R7. We then examined Dpn expression in *sev*.sev* eye discs in which Sev is ectopically active in cone cell precursors, and observed the corresponding ectopic expression of Dpn in those cells (Fig 5C). From these results we infer that the expression of Dpn is intimately associated with high-level transduction of the RTK pathway.

#### Phyl is required for Dpn expression

*phyl* is the target gene of the RTK pathway, and we asked whether Dpn expression is mediated by *phyl* transcription or by some other target of the pathway. To abrogate *phyl* gene function we expressed a *phylRNAi* transgene using *GMR*.*Gal4* and observed normal R3/4 Dpn staining but its loss from R7 precursors (Fig 5E). Furthermore, when *phyl* expression was restored to *sev°* eyes using a *sev*.*phyl* transgene, Dpn expression was restored to the R7 precursor (Fig 5D). Ostensibly, these results suggest that Phyl is necessary and sufficient for mediating the role played by the Sev pathway in regulating Dpn expression in the R7 precursor. However, in the Discussion we revisit this point and offer a subtly different interpretation.

#### Removal of Ttk restores Dpn expression

Since Phyl acts to degrade Ttk, we next asked whether the Phyl regulation of Dpn is mediated through Ttk removal. In the *GMRG4-phylRNAi* background described above, Dpn expression is lost from R7 precursors (Fig 5E), but when we additionally included a *ttkRNAi* transgene, Dpn expression was restored (Fig 5F). Thus, the effects on Dpn expression caused by the loss of Phyl are reversed if Ttk is concomitantly lost. This argues that when Phyl is lost, it is the persistent Ttk that prevents Dpn expression, and we infer that Dpn expression is normally repressed by Ttk.

### Ttk represses *dpn* transcription

**Fig 6:**
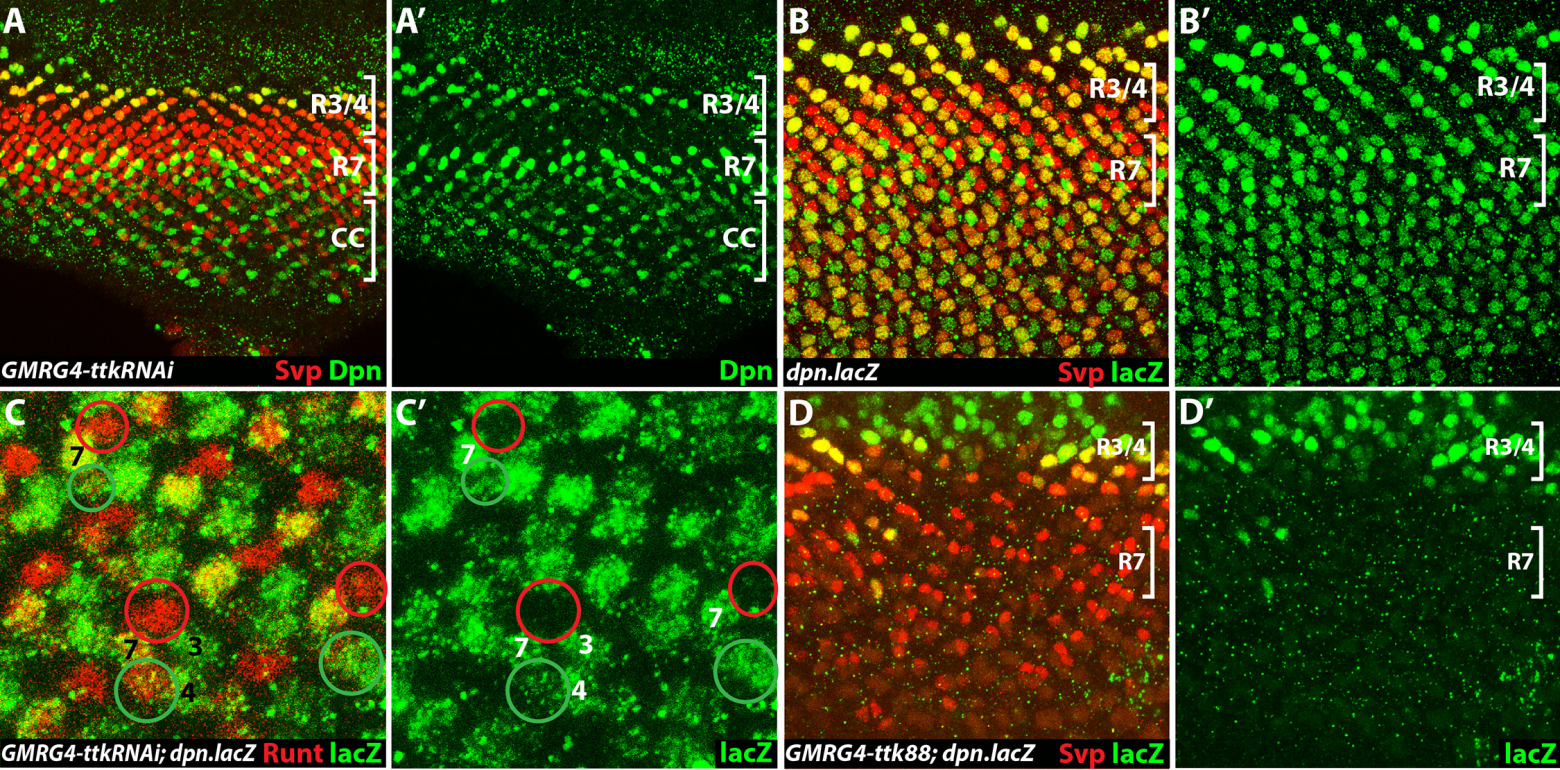
Ttk regulates Dpn expression. (**A, A’**) *GMR-ttk*.*RNAi* eye discs show the normal R3/4 and R7 expression of Dpn, but later ommatidia also show staining in cone cell precursors (cc—lower bracket). (**B, B’**) *dpn*.*lacZ* expression (green) shows a similar R3/4 and R7 staining pattern to the Dpn protein except that the expressions are long-lived accounting for the extensive staining seen in the more mature ommatidia (below the R7 stage in the panel). (**C, C’**) Image shows the posterior region of a *GMR*.*ttkRNAi/dpn*.*lacZ* eye discs stained for LacZ (green) and for the R7 marker Runt (red). *dpn*.*lacZ* expression occurs selectively in posterior and not anterior cone cells. The R7s are numbered and express Runt and LacZ. Flanking them are the Anterior (AC) and Posterior (PC) cone cells (circled red and green respectively). Both these cone cell precursors express Runt, indicative of the adoption of the R7 fate, but PC also expresses LacZ whereas AC does not. (**D, D'**) Ttk suppresses *dpn*.*lacZ* expression. D shows an image through a *GMR-ttk88/dpn*.*lacZ* labeled for lacZ (green) and Svp (Red). Although the R3/4 staining (upper bracket) appears normal, staining at the R7 levels (lower bracket) is absent.

When we examined Dpn expression in *GMR-ttkRNAi* eye discs we saw the normal expression pattern (including R7s) but additionally observed ectopic staining in the cone cell precursors in the more mature ommatidial clusters (cc, Fig 6A). This argues that removal of *ttk* leads to ectopic expression of Dpn, and we next asked whether Ttk regulates *dpn* transcription. We began by examining the expression pattern of the *dpn*.*lacZ* transgene (Fig 6B), and observed that expression began in the R3/4 and R7 precursors normally, but did not extinguish and persisted into the more mature ommatidial clusters. This reporter appears as a faithful indicator of when *dpn* transcription is initiated, but whether the later staining is indicative of normal *dpn* transcription (which we doubt) or whether it is artifactual (perdurance of the *lacZ* gene product for example) remains unclear. We introduced *GMR-ttkRNAi* into this background and observed the additional ß-galactosidase staining in cone cell precursors (Fig 6C). The anterior (AC) and posterior (PC) cone cells are the first two cone cells recruited, and the presence of *GMR-ttkRNAi* re-specifies them both as R7s (as evidenced by Runt staining—red). Interestingly, the ectopic ß-galactosidase staining occurred in PC and not AC (Fig 6C'). In the next section of the paper we define a critical role for N function in *dpn* transcription, and note here that N activity terminates first in AC and we suspect that it is the low N activity in this cell that prevents *dpn*.*lacZ* expression, and we revisit and expand upon this in the Discussion. The gain of *dpn*.*lacZ* expression in cone cells argues that Ttk regulates Dpn expression at the level of transcription. Indeed, if Ttk functions to repress *dpn* transcription, then the forced expression of Ttk would be expected to prevent the expression of *dpn*.*lacZ* in R7 precursors. To this end, we engineered and transformed a *UAS*.*ttk88* transgene and expressed it with *GMR*.*Gal4* in the *dpn*.*lacZ* background (Fig 6D). Here we observed the loss of ß-galactosidase staining from the R7 regions of the eye discs (lower bracket) but saw a normal expression at the R3/4 level (upper bracket). Collectively, from the effects of loss and gain of Ttk activity, we infer that Ttk functions to repress *dpn* transcription.

### Notch activity regulates Dpn expression

The results above suggest that Ttk degradation is required for the expression of Dpn. But if removal of Ttk were all that was needed for Dpn expression, then following Ttk removal we would expect chronic expression of Dpn in the R7 precursor. This does not happen; the Dpn expression in R7 precursors is short lived. Furthermore, Ttk is degraded in R1/6 precursors and these cells do not express Dpn. We therefore inferred the action of another agency, and since the N pathway is active in the R7 precursor, and ectopic N activation in the eye had already been shown to induce ectopic Dpn expression [11], we began a detailed investigation of the role of N in Dpn regulation.

#### High-level N activity in R1/6 precursors elicits Dpn expression

**Fig 7:**
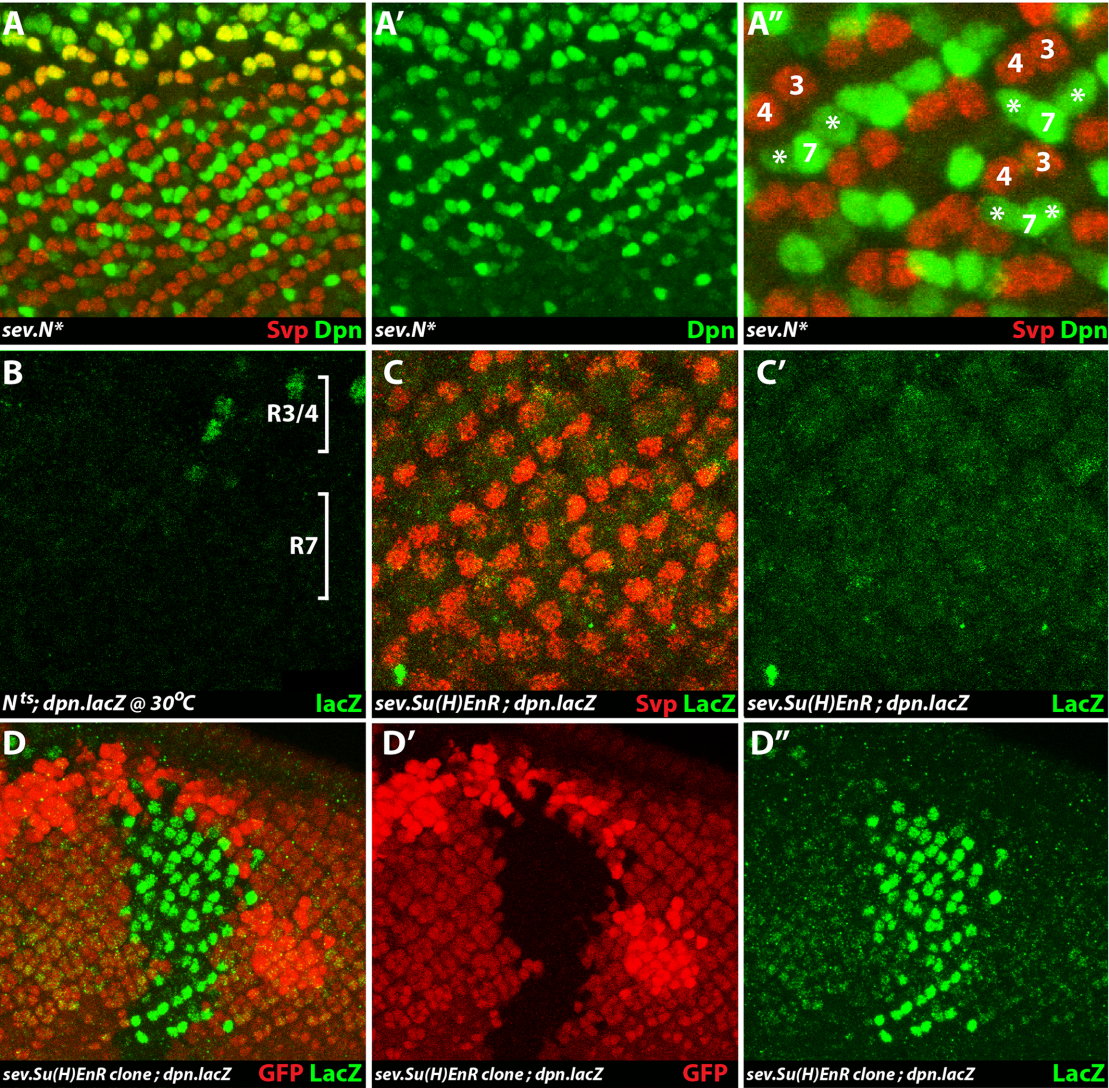
N activity promotes *dpn* transcription. (**A, A’, A”**) Ectopic N activation induces ectopic Dpn expression. When N is activated in R1/6 precursors (*sev*.N*) they no longer express Svp (red) and ectopically express Dpn (green). (**A”**) shows a high power image to highlight the R1/6 precursors expressing Dpn (asterisks). (**B**) When *dpn*.*lacZ* activity (green) is monitored in an eye disc of *N*^*ts*^ animals raised for 24hrs at 30°C, there is residual staining in some R3/4 precursors, but no staining is seen in the R7 positions. (**C, C’**) In *sev*.*Su(H)EnR* eye discs, Svp expression (red) indicates that R3/4 and R1/6 precursors are patterned normally but there is an absence of *dpn*.*lacZ* expression (green) from all R3/4 and R7 precursors. (**D, D’, D”**) When a wild type clone (black) is induced in a *sev*.*Su(H)EnR* background (red), the *dpn*.*lacZ* expression in R3/4 and R7 precursors is significantly stronger.

When an activated form of N (N*) is expressed under *sev* transcriptional control (*sev*.N*), high-level N activity is supplied to R1/6 precursors, and staining of such *sev*.N* eye discs revealed ectopic Dpn expression in these cells (Fig 7A). Although the R7 and R1/6 precursors form a triumvirate of cells, the presence of sev.N* frequently leads to the loss of one of the three cells, and this accounts for why many of the ommatidia show only two Dpn-positive cells in the R1/6/7 positions. These results argue that once Ttk is degraded (as happens normally in R1/6 precursors) and N activity is concomitantly high, then Dpn is expressed.

#### Reduced N activity abrogates *dpn* transcription

We next used the *dpn*.*lacZ* reporter line to evaluate whether N activity promotes *dpn* transcription. First we negated N gene function by shifting third instar larvae of the N temperature sensitive allele (*N*^*ts*^) to the restrictive temperature for 24hrs, and then examined *dpn*.*lacZ* expression in the eye discs (Fig 7B). Here ß-galactosidase activity was removed from both the R3/4 and R7 regions, suggesting that *dpn* transcription is under N control in both cell types. We next expressed a dominant suppressor of N target genes (the Suppressor of Hairless transcription factor fused to the Engrailed repressor domain) under *sev* transcriptional control (*sev*.*Su(H)enR*). When *dpn*.*lacZ* was monitored in such *sev*.*Su(H)enR* eye discs, there was a dramatic reduction of ß-galactosidase in both the R3/4 and R7 precursors (Fig 7C). When *sev*.*Su(H)enR* was removed in a clonal manner (black region in Fig 7D) there was a corresponding and strong increased *dpn*.*lacZ* expression (green) in R3/4 and R7 precursors (Fig 7D). Collectively these results argue that N activity is critically required for *dpn* transcription in R7 (and R3/4) precursors.

## Discussion

The Drosophila eye is an exquisitely structured photoreceptive organ that requires a precisely orchestrated developmental program to specify the appropriate cells in the correct position, and in this study we examine the mechanisms that ensures that an R7 photoreceptor is generated in every ommatidium. Within the R7 developmental program, the Boss ligand and Sev RTK are engaged to provide a requisite high-level activation of the RTK pathway which promotes the expression of Phyl and elicits the Ubiquitination and degradation of Ttk. Since Ttk is the transcription factor that represses the photoreceptor fate, the RTK pathway plays an indispensible role in specifying the R7 precursor as a photoreceptor. Indispensible as it is, expression of Phyl alone does not appear to be sufficient for the specification of all R7s, since abrogation of *dpn* gene function leads to ommatidia in which R7s are absent. We find here that Dpn functions to repress transcription from the *ttk* locus, and cooperates with the degradation mechanism in Ttk removal. We note that Dpn expression ceases early in the life of the R7 precursor and does not therefore function as a long-term mechanism to prevent the resupply of Ttk. Rather, we see it as acting coincidentally with the degradation mechanism, with both required to achieve effective Ttk elimination, and the resulting unambiguous specification of the R7 photoreceptor fate. Both the N and RTK pathways that converge in this mechanism are also short lived, and it remains unclear how the clearing of Ttk from the R7 precursor locks the cell into the photoreceptor pathway.

Dpn is both expressed and required in the R7 precursor, and an elegant mechanism utilizing the combined action of the RTK and N pathways ensures that it is selectively expressed in this cell. First we will address the part played by Ttk and argue that it performs two different roles. The primary role of Ttk is to repress the photoreceptor fate (through the regulation of unknown target genes). The secondary role is to prevent the expression of Dpn, and thereby prevent a reduction in its own transcription. This is the function that it plays in cone cell precursors, where in silencing Dpn it prevents the reduction of its own levels that would make it vulnerable to the low-level RTK activity in those cells. Cone cells and nascent R7s are identical in this regard, but as the high level RTK pathway operates in the R7 precursor, a dramatic change ensues resulting in the expression of Dpn and the shutting down of *ttk* transcription. However, in the cone cell precursors nothing changes, and Ttk persists in repressing Dpn.

The other key aspect of Dpn regulation is the role played by N. Consider the R1/6 precursors; they degrade Ttk (and thereby become photoreceptors) but do not express Dpn. Hence, the simple removal of Ttk is insufficient for the expression of Dpn; high-level N activity is also required. N activity is low in R1/6 precursors but high in the R7 (and cone cell) precursors, where it provides the promotion of Dpn expression. Only in the R7 precursor are the requisite conditions for Dpn expression achieved; the repressor (Ttk) is removed, and the promoter (high N activity) is present.

Mutually repressive transcription factor interactions normally resolve to one of two bi-stable states in which one dominates the other. In the R7 precursor we can see how a switch between two such states can be regulated by signals entering the cell. In the nascent R7s, Ttk levels are high and the *dpn* locus is silent. But this situation is altered once the RTK pathway begins Ttk degradation. As the level of Ttk drops below a certain threshold, *dpn* transcription initiates, and the resulting Dpn protein then feeds back to silence transcription from the *ttk* locus. Thus the RTK pathway acts to switch the Ttk/Dpn relationship from Ttk-dominant to Dpn-dominant, and in doing this, the clearance of Ttk protein required for photoreceptor specification is achieved.

The Enhancer of Split genes are targets of the N pathway, and a number of them encode transcription factors of the helix-loop-helix class. These transcription factors mediate many N activities, but their expression patterns are poorly understood in the developing eye, and which ones mediate N functions in R7 specification has been difficult to determine. However, an enhancer element from the *Mdelta* gene is expressed selectively in the R7 precursor (not in the R1/6 or cone cell precursors) and ectopic expression of this gene in R1/6 precursors re-specifies them as R7 types (albeit at a modest frequency)[15, 16]. Since Dpn is closely related to the E(Spl) family of proteins, we suggest that it likely has the same ability to re-specify R1/6 precursors as R7s. This begs the question of whether Dpn normally performs this function in R7, or whether it mimics the activity of one or more of the E(Spl) proteins. We cannot currently answer this question, but we note that Dpn is expressed in R7 and not R1/6 and so has the spatial restriction needed to perform that function. We also note that *dpn* loss of function does not switch the fate of R7s to R1/6s (although this may simply result from redundancy with other factors).

Although we remain agnostic on the question of whether Dpn functions to specify the R7 photoreceptor type, the fact that ectopic Dpn expression in R1/6 precursors changes their fate, highlights the fact that are now two reasons why Dpn expression is exclusive to R7 precursors in second wave cells. First, as described above, if it were expressed in cone cell precursors, their lowered Ttk levels might lead them to be specified as photoreceptors. Second, we now see that although Dpn expression in R1/6 precursors would not disturb their specification as photoreceptors (their Ttk is already degraded), it would disturb which type of photoreceptor they would be specified as.

When Ttk activity is reduced in cone cell precursors (*GMRG4*.*ttkRNAi*), although both the anterior and posterior cone cells (AC and PC) were (as expected) re-specified as R7s (as evidenced by Runt expression) there was a striking asymmetry in the de-repression of *dpn*.*lacZ* activity; it occurred in PC and not AC. We infer that this relates to the different N activities in these two cells. Sev expression is a simple meter of N activity in the R7 equivalence group [6], and we noticed many years ago that Sev staining decayed in AC before PC [17]. From this we infer that N levels fall first in this cell, and since *dpn*.*lacZ* critically requires high N activity for its expression, the failure to de-repress *dpn*.*lacZ* in AC simply results from the absence of the requisite high N activity.

In addition to the roles played by Ttk and N in regulating Dpn, we also infer an additional (Phyl-independent) role played by the RTK pathway. Consider the expression of Phyl in a *sev* mutant background. In one experiment we show that this is not sufficient to restore all R7s (Fig 2C), requiring the co-expression of Dpn (Fig 2E) for full rescue. Yet, in another experiment we show that expression of Phyl in a *sev* mutant background promotes that requisite Dpn expression (Fig 5D). The simplest explanation here is that the RTK pathway promotes *dpn* transcription through two independent mechanisms, and only when both are active is a sufficient level of Dpn generated. One mechanism is the process we have described in which RTK activity promotes *phyl* expression that results in the removal of Ttk. The other mechanism is unknown, but one possibility is that the *dpn* gene is a direct target of the RTK pathway. In this scenario we envisage the RTK pathway to directly promote *dpn* transcription, and to indirectly de-repress its expression through promotion of *phyl* transcription.

There is an enigma at the heart of N activity in the R7 precursor; it both promotes and opposes the removal of Ttk from the cell. This enigma likely reflects the evolutionary history of the ommatidium, and suggests that the role of N in opposing Ttk removal represents an ancient function designed to prevent the promiscuous specification of photoreceptors [18], and that in repurposing the cell in the R7 position to become a photoreceptor, the high N activity was used to activate new mechanisms to counteract the primary function. We previously described the transcription of *sev* as one of these new mechanisms, and now recognize *dpn* transcription as another. Indeed, we now list four distinct N functions in the R7 precursor; the ancient role that opposes Ttk removal, the two functions that promote Ttk elimination (*sev* and *dpn* transcription), and the role that specifies the R7 versus the R1/6 fate once Ttk is degraded. All four functions occur roughly simultaneously, highlighting the impressive, and counterintuitive ways that N is employed in R7 specification, and the R7 photoreceptor is emerging not just as a classic model for studying cell specification, but also becoming as an excellent system to probe a cell’s evolutionary history.

## Materials and Methods

### Immunohistochemistry and histology

Protocols for adult eye sectioning and antibody staining have been described previously [6, 19]. In all data analyses the R8 photoreceptors were individually identified to ensure that they were not mistaken as supernumerary R7s. Primary antibodies: rabbit anti-β galactosidase (Cappel); rabbit anti-GFP, mouse anti-GFP IgG2a (Molecular Probes); guinea pig anti-Runt (gift of J. Reinitz, Stony Brook University, NY, USA); rabbit anti-Runt (gift of Andrea Brand, Gurdon Institute, Cambridge, UK); guinea pig anti-Dpn (gift of James Skeath, Washington University School of Medicine, Washington, USA); mouse anti-Svp (gift of Y. Hiromi, National Institute of Genetics, Japan); rat anti-Elav, mouse anti-Cut (Developmental Studies Hybridoma Bank). Alexa Fluor 488, 555, 647 conjugated secondary antibodies were used (Molecular Probes). For amplification of the β-galactosidase staining, Donkey anti-rabbit HRP conjugated secondary antibodies were used. Amplification was conducted using Tyramide Signal Amplification (TSA) systems (PerkinElmer) following manufacturer’s protocol.

### Fly stocks

*sev[d2]*, *sev*.*N[act]*, *sev*.*sev[act]*, *N*^*ts*^, *sev*.*Su(H)enR*, *GMR*.*sev*, *sev*.*phyl*, *ttk*.*lacZ*, *sev*.*Gal4*, *GMR*.*Gal4*, (see [6, 20]. *dpn1*, *UAS*.*dpn* (gifts of Sarah Bray), *UAS ttkRNAi* [TRiP stock HMS03008], *UAS dpnRNAi* [TRiP stock HMC03154] (Bloomington Stock Centre). *UAS phylRNAi* [GD12579 v35469] VDRC, *>tub*.*Gal80>* (gift of Gary Struhl).

### Generation of clones

*Sev*.*Su(H)EnR* clones were induced as previously described[6]. Clones expressing *sevG4-dpn* were induced in a *hs*.*flip; >tub*.*Gal80>/dpn*.*lacZ; Sev*.*Gal4/UAS*.*dpn* background by 37°C heat shocks given to larvae at 24-48hrs AEL. *dpn1* clones were generated in *w*,*hs*.*flip; FRT42D*, *dpn1/FRT42D*, *w*^*+*^, *M(2)53C* flies by 37°C heat shocks delivered 24-72hrs AEL.

### N^ts^ experiments

N^ts^ flies were reared at 18°C and shifted to 30°C in the third instar for 24 h followed by dissection and immunohistochemistry.

### Generation of *UAS*.*ttk88*

The coding sequence for *ttk88* was amplified from the cDNA provided by Craig Montell (John Hopkins University School of Medicine) using appropriate PCR primers and cloned into the attB UAS vector using KpnI-EcoRI restriction enzymes, and transformed using standard protocols.
